# Supplementary material for: A Global Screen for Assembly State Changes of the Mitotic Proteome by SEC-SWATH-MS
Source: Cell Syst. 2020 Feb 26;10(2):133–155.e6. doi: 10.1016/j.cels.2020.01.001 (PMC7042714; doi:10.1016/j.cels.2020.01.001)

P61221 | ABCE1_HUMAN | ABCE1 RLI RNASEL1 RNASELI RNS4I OK/SW−cl.40
Monomer MW [kDa]: 67.314 Monomer expected elution fraction: 44

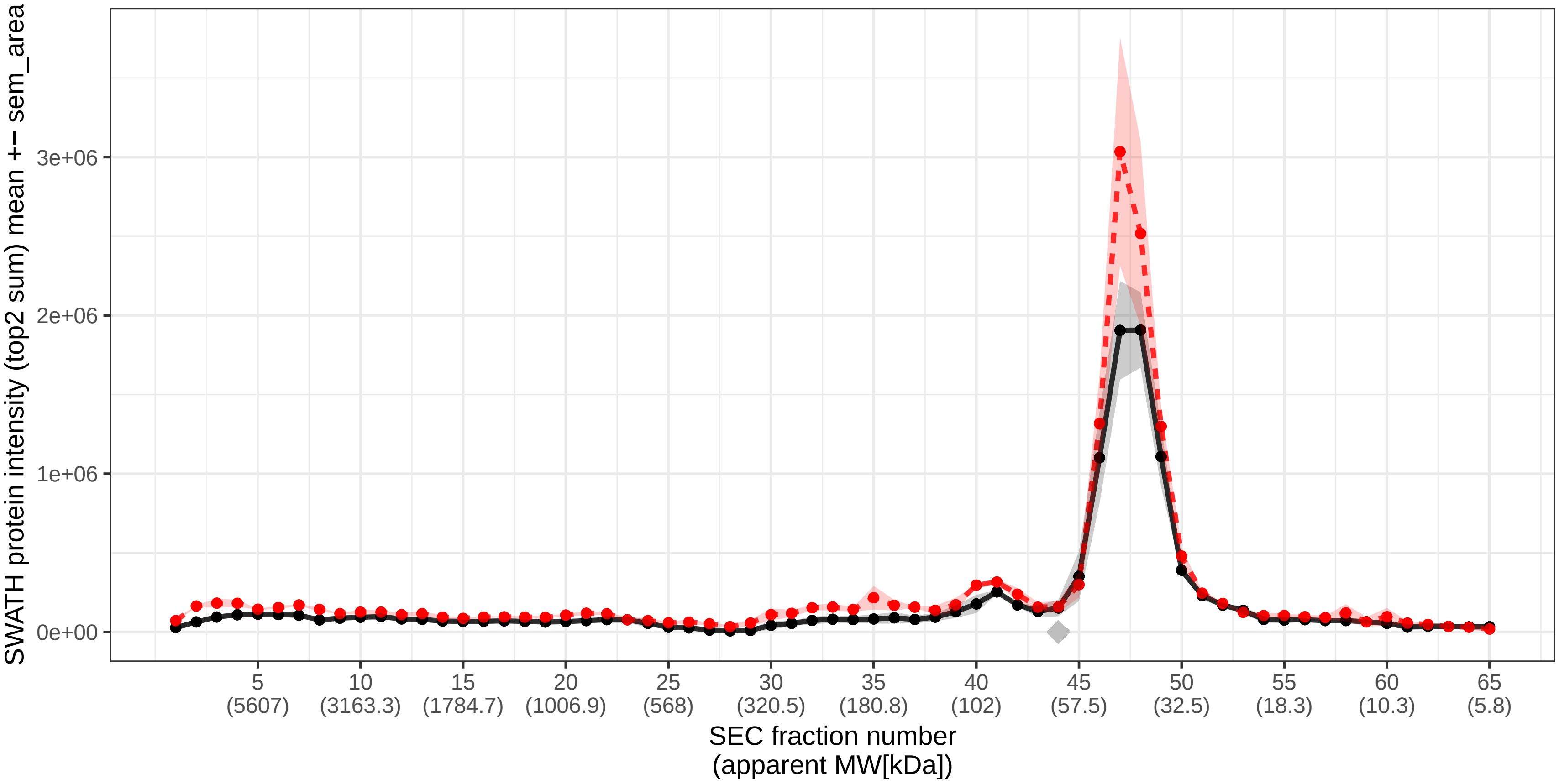

Supplement: Data S1. SEC-SWATH-MS Protein Chromatograms, Related to Figure 1 [file mmc6.zip › SECchrom_P61221_ABCE1_HUMAN_ABCE1_RLI_RNASEL1_RNASELI_RNS4I.pdf]
